# Effects of Free or Immobilized *Pediococcus acidilactici* ORE5 on Corinthian Currants on Gut Microbiome of Streptozotocin-Induced Diabetic Rats

**DOI:** 10.3390/microorganisms12102004

**Published:** 2024-10-02

**Authors:** Ioanna Prapa, Vasiliki Kompoura, Chrysoula Pavlatou, Grigorios Nelios, Gregoria Mitropoulou, Nikolaos Kostomitsopoulos, Stavros Plessas, Eugenia Bezirtzoglou, Vaios T. Karathanos, Amalia E. Yanni, Yiannis Kourkoutas

**Affiliations:** 1 Laboratory of Applied Microbiology and Biotechnology, Department of Molecular Biology and Genetics, Democritus University of Thrace, Dragana, 68100 Alexandroupolis, Greece; ioannaprap@gmail.com (I.P.); vickykom20.70@gmail.com (V.K.); xrysapavl@gmail.com (C.P.); gregnelios@hotmail.com (G.N.); grigoriamitropoulou@gmail.com (G.M.); 2 Laboratory Animal Facility, Biomedical Research Foundation of the Academy of Athens, 11527 Athens, Greece; nkostom@bioacademy.gr; 3 Laboratory of Microbiology, Biotechnology and Hygiene, Faculty of Agricultural Development, Democritus University of Thrace, 68200 Orestiada, Greece; splessas@agro.duth.gr; 4 Laboratory of Hygiene and Environmental Protection, Department of Medicine, Democritus University of Thrace, Dragana, 68100 Alexandroupolis, Greece; empezirt@med.duth.gr; 5 Laboratory of Chemistry, Biochemistry, Physical Chemistry of Foods, Department of Nutrition and Dietetics, Harokopio University of Athens, 17671 Athens, Greece; vkarath@hua.gr; 6 Agricultural Cooperatives’ Union of Aeghion, Corinthou 201, 25100 Aeghion, Greece

**Keywords:** functional foods, gut microbiome, type-1 diabetes, *Pediococcus acidilactici*, Corinthian currants, cell immobilization, presumptive probiotic

## Abstract

The present study aimed to investigate the effect of a dietary intervention including free or immobilized cells of the presumptive probiotic *Pediococcus acidilactici* ORE5 on Corinthian currants, a food with beneficial impact in the condition of Type-1 Diabetes Mellitus (T1DM), on the microbiome composition of STZ-induced diabetic rats. Twenty four male Wistar rats were divided into four groups (*n* = 6 per group): healthy animals, which received the free (H_FP) or the immobilized *Pediococcus acidilactici* ORE5 cells (H_IPC), and diabetic animals, which received the free (D_FP) or the immobilized *Pediococcus acidilactici* ORE5 cells(D_IPC) for 4 weeks (10^9^ cfu/day, in all groups). At the end of the dietary intervention, the D_IPC group exerted a lower concentration of the inflammatory cytokine IL-1 beta compared to D_FP. Consumption of immobilized *P. acidilactici* ORE5 cells on Corinthian currants by diabetic animals led to increased loads of fecal lactobacilli and lower *Enterobacteriaceae*, coliforms, and *Escherichia coli* levels, while Actinobacteria phylum, *Akkermansia*, and *Bifidobacterium* genera abundances were increased, and fecal lactic acid was elevated. Overall, the results of the present research demonstrated that functional ingredients could ameliorate gut dysbiosis present in T1DM and could be used to design dietary patterns aiming at T1DM management. However, well-designed clinical trials are necessary, in order to confirm the beneficial effects in humans.

## 1. Introduction

Nowadays, the elucidation of the causal relationship between the intestinal microbiome and the onset and development of pathological conditions is an area of focused research. Type-1 Diabetes Mellitus (T1DM), characterized by autoimmune destruction of pancreatic beta cells, is a condition influenced by the gut microbiota [1,2]. In 2021, it was estimated that around 8.4 million people were facing T1DM globally, including the 500,000 new cases reported during that year. According to provisions, the number of people suffering from T1DM will range between 13.5 and 17.4 million by 2040 [3].

A variety of factors that have become increasingly common nowadays appear to play a role in the rising incidence of T1DM (lack of breastfeeding, method of birth delivery, childhood dietary patterns, antibiotic utilization, exposure to microbes, etc.). The manipulation of the microbiome could influence the impact of these factors on the susceptibility to T1DM [4]. Additionally, emerging research suggests that disturbances in the gut microbiota might play a role in the development of T1DM, with gut dysbiosis potentially leading to immune dysregulation and increased intestinal permeability as potential mechanisms underlying the onset of T1DM [2,5].

Amidst the strategies for managing the disease of T1DM, probiotics have emerged as a promising agent for modulating the gut microbiome and potentially impacting disease progression [6]. Findings from both experimental research and clinical trials suggest that probiotic administration could offer a protective effect against T1DM through various mechanisms. For example, probiotics have been shown to enhance the expression of junction and adhesion proteins, bolstering the integrity of the gut epithelial barrier [7]. Additionally, they can lower oxidative stress, regulate inflammatory responses, and consequently promote the proliferation of T-regulatory cells and the production of anti-inflammatory cytokines, all of which contribute to their potential protective effects against T1DM [8,9]. However, while numerous studies have explored the effects of probiotics on gut health and metabolic disorders, the efficacy of novel wild-type presumptive strains particularly in the context of T1DM, needs to be examined separately.

Cell immobilization on natural food ingredients is proposed as an easy and natural technique to produce food ingredients with enhanced shelf-life and cell survival during storage and digestion [10,11]. Combining a probiotic culture with a food constituent that has beneficial properties could lead to the production of a functional food ingredient or a presumptive synbiotic. As natural immobilization agents, fruit pieces, nuts, and cereals, among others, have been previously tested successfully [10,11]. Corinthian currants are dried grapes rich in polar phenolic compounds [12], which have shown beneficial effects in streptozotocin (STZ)-induced diabetic rats (T1DM). Specifically, after a 4-week dietary intervention, serum polar phenolic compounds were restored, levels of the inflammatory factor IL-1 beta were reduced, and the intestinal microbiome composition was balanced [13].

Novel wild-type probiotic strains are vastly needed given the increasing trend of the probiotic industry, in recent years. *Pediococcus* spp. are lactic acid bacteria (LAB), and selected strains have been characterized as probiotics. *Pediococcus acidilactici* ORE5, isolated from kefir grains, has been evaluated for potential probiotic properties in vitro by our research group [14]. Particularly, the survival of the strain after exposure to low pH, pepsin, pancreatin, and bile salts, along with sensitivity to antibiotics, indicated the fitting of the strain as a probiotic candidate. Moreover, the incorporation of the strain in Katiki Domokou-type cheese ameliorated the cheese microbiome [14].

To the best of the authors’ knowledge, there are only a few reports in the literature that have investigated the effects of a dietary supplementation with presumptive probiotics on microbiome composition in T1DM rats (few of them with synbiotics and none with immobilized cells). Hence, the aim of the present study was to investigate the potential alterations in the microbiome composition of STZ-induced diabetic rats after a 4-week dietary intervention with immobilized cells of the presumptive probiotic *P. acidilactici* ORE5 on Corinthian currants (functional food ingredient) in comparison to free cells. The effects on blood parameters related to the disease of T1DM were also examined.

## 2. Materials and Methods

### 2.1. Microbial Strain

*Pediococcus acidilactici* ORE5, isolated from kefir grains [14], was grown on Man, Rogosa, and Sharpe (MRS) broth (Condalab, Madrid, Spain) at 37 °C for 24 h. The medium was sterilized at 121 °C for 15 min prior to use.

### 2.2. Immobilization of Cells on Corinthian Currants and Freeze-Drying

Cell immobilization on Corinthian currants (*Vitis vinifera* L., var. Apyrena, “VOSTIZZA” Protected Designation of Origin, P.D.O., kindly provided by the Agricultural Cooperatives’ Union of Aeghion, Aeghion, Greece) was carried out as described previously [11]. Before the initialization of the immobilization process, Corinthian currants were heated at 120 °C for 10 min as a pretreatment to eliminate indigenous microbes. Then, 500 mL of the grown *P. acidilactici* ORE5culture were centrifuged (8500× *g*, 15 min, 4 °C), washed with 100 mL of sterile ¼ Ringer’s solution, and resuspended in 250 mL ¼ Ringer’s solution. In this cell suspension, 200 g of Corinthian currants, used as immobilization support, were added, and the mixture was left undisturbed for 6 h at room temperature (RT) to achieve cell immobilization, as cells are attached to the surface and at the inner part of the food matrix. Thereafter, the immobilized cells (on Corinthian currants) were strained and washed with sterile ¼ Ringer’s solution, in order to remove non-immobilized cells.

Immobilized *P. acidilactici* ORE5 cells were transferred to −80 °C for 18 h and then freeze-dried on a BenchTopPro freeze-dryer (Virtis, SP Scientific, Warminster, PA, USA) for 24 h (30–35 Pa vacuum and −101 °C condenser temperature). Cells before and after freeze-drying were stored at RT and at 4 °C and cell survival was monitored for up to 30 days. For comparison purposes, freeze-dried free (non-immobilized) *P. acidilactici* ORE5 cells were also prepared.

### 2.3. Scanning Electron Microscopy

To verify cell immobilization of *P. acidilactici* ORE5 on Corinthian currants, scanning electron microscopy was performed, as described recently [10]. Briefly, gold was used to coat freeze-dried immobilized cells with a coating system (BAL-TEC MED 020 Sputter, BAL-TEC, Balzers, Liechtenstein) for 2 min, following examination using a JSM-6300 scanning electron microscope (Jeol Ltd., Tokyo, Japan) at 20 kV voltage.

### 2.4. In Vivo Study Design

#### 2.4.1. STZ-Induced Animal Model

Twenty-four male Wistar (RCCHan:Wistar) rats (Envigo, Italy) bred in the Laboratory Animal Facility of the Biomedical Research Foundation of the Academy of Athens (BRFAA) were employed in the study. Power analysis using the software “G*Power 3” was used to determine the number of animals that were needed to draw significant conclusions [15]. F-test analysis of variance (ANOVA) was performed using data from a pilot study [16], also reported elsewhere [13]. Based on the results, 7 animals per group were entailed. Given the losses observed after STZ injection (~25%), each group finally included 6 animals, which has been proven to be a sufficient number to draw significant differences, as demonstrated in previous studies [13,16].

One week before protocol debut, rats were housed individually and had ad libitum access to food (4RF22, Mucedola, Udine, Italy) and tap water (acclimatization period). For the induction of diabetes, 14-week-old rats that weighted 360–380 g were employed, in accordance with our previous study [13]. Animals in a non-fasting state were injected with 60 mg/kg of body weight STZ (Sigma-Aldrich, Darmstadt, Germany) solution (STZ in citrate buffer 0.1 M, pH 4.5) intraperitoneally. One week post-injection, blood glucose concentration was determined as described below, and animals exerting levels ≥ 250 mg/dL [17] were considered diabetic and were included in the study. All animal experimentations were conducted according to the European Directive 2010/63, and protocols were reviewed and approved by the Veterinary Directorate of the Athens Prefecture (Ref. Number 272253/07-04-2021). Animal well-being was monitored and supervised by a designated veterinarian of the BRFAA animal facility. The animals were individually housed, in order to reassure that they received the respective dietary regimen daily dosage. Every week, bedding and cages were changed. Body weight of the animals was measured weekly, and blood glucose was monitored using a digital glucose meter (OneTouch Verio FlexTM, Lifescan Ltd., Malvern, PA, USA) to confirm the establishment and maintenance of T1DM.

#### 2.4.2. Dietary Intervention with Probiotics

**Figure 1:**
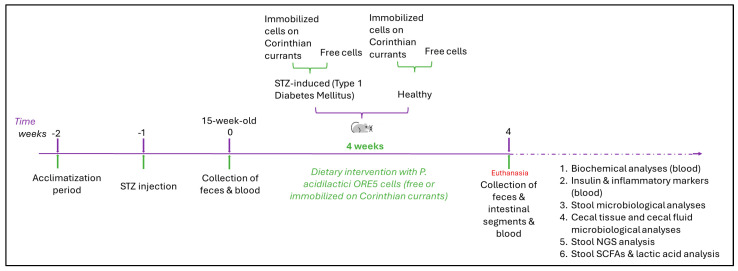
Schematic flowchart of the in vivo experimental design.

Animals were divided into 4 groups according to their diet (*n* = 6 per group): healthy animals that received the control diet supplemented with 10^9^ cfu freeze-dried immobilized *P. acidilactici* ORE5 cells on Corinthian currants (H_IPC), or diabetic animals that received the control diet supplemented with 10^9^ cfu freeze-dried immobilized *P. acidilactici* ORE5 cells on Corinthian currants (D_IPC), healthy animals that received the control diet supplemented with 10^9^ cfu free *P. acidilactici* ORE5 cells (H_FP), or diabetic animals that received the control diet supplemented with 10^9^ cfu free *P. acidilactici* ORE5 cells (D_FP). Both diets were isocaloric, and Corinthian currants represented 10% (*w*/*w*) of the diet [13]. Every day, the animals were provided with fresh food, and any remains were removed. The dietary intervention lasted for 4 weeks (28 days). A schematic flowchart of the groups and the in vivo study design is presented in Figure 1.

#### 2.4.3. Sample Collection

At baseline and at the 4th week of the study, blood and stool samples were collected. Blood was obtained from the lateral tail vein of the animals after a six-hour fasting, and heparinized plasma was isolated following centrifugation (3000× *g*, 10 min, 4 °C). At the end of the experimental period, animals were anesthetized by an overdose of isoflurane (ISOVET, Chanelle Pharma, Loughrea, Co., Galway, Ireland), followed by euthanasia by exsanguination after the collection of blood from the anterior vena cava. The intestinal segment of cecum and the cecal content (fluid) were also collected. Cecal tissue was washed twice with normal saline and diluted (1:1) in 25% glycerol-Ringer’s solution. All samples (plasma, stools, cecum tissue, and cecal fluid) were stored at −80 °C until analysis.

### 2.5. Sample Analyses

#### 2.5.1. Blood Analyses

Plasma glucose, total cholesterol (TC), and triglycerides (TG) were assessed on an automated biochemical analyzer (Konelab 60i, Thermo Fisher Scientific Inc., Waltham, MA, USA) using commercially available kits (Thermo Fisher Scientific Inc.). Plasma insulin levels were determined by a rat insulin enzyme-linked immunosorbent assay (ELISA) kit (EZRMI-13K, Merck Millipore, Darmstadt, Germany). Inflammatory factors Tumor necrosis factor-a (TNF-a), Interleukin-1 beta (IL-1 beta), and Interleukin-6 (IL-6) were quantified in samples derived from the cardiac puncture at the end of the dietary intervention using ELISA kits (OriGene, Rockville, MD, USA).

#### 2.5.2. Microbiological Analyses

##### Determination of *P. acidilactici* ORE5 Levels

Cell levels of *P. acidilactici* ORE5 (free or immobilized on Corinthian currants, wet or freeze-dried) were determined after serial decimal dilutions on sterile ¼ Ringer’s solution and plating on MRS agar, as described below. Prior to analysis, freeze-dried immobilized cells were rehydrated in sterile dH_2_O for 1 h at RT, followed by filtration. Five g of immobilized cells (wet or rehydrated freeze-dried) were homogenized with 45 mL of sterile ¼ Ringer’s solution, followed by serial decimal dilutions in the same solution and plating on MRS agar (Condalab) at 37 °C for 72 h anaerobically [Anaerobic Jar 2.5 L (Merck Millipore), Anaerocult A system (Merck-Millipore)]. Likewise, free cell biomass was rehydrated in sterile dH_2_O corresponding to the initial volume (before freeze-drying) at RT for 15 min. The % survival rate was calculated by dividing the cell loads expressed as logcfu/g or logcfu/mL at each timepoint by the initial logcfu/g or logcfu/, and multiplied by 100, as previously described [11].

##### Stool and Tissue Microbiota Analyses

Fecal samples (1–2 g), cecal intestinal fluid (2–3 g), or intestinal (cecum) tissue (2–3 g) were vigorously blended with 0.1% sterilized buffered peptone water (Lab M, Heywood, UK) to achieve homogenization. After serial decimal dilutions on sterile ¼ Ringer’s solution, pouring/plating on different media was followed to determine: (1) Total Aerobic Counts (TAC) on Plate Count agar (Condalab) at 30 °C for 72 h, (2) *Enterobacteriaceae* on Violet Red Bile Glucose agar (Condalab) at 37 °C for 24 h, (3) coliforms on Chromogenic Coliform Agar (Condalab) at 37 °C for 24 h, (4) *Escherichia coli* on Tryptone Bile-X Chromogenic agar (Condalab) at 37 °C for 24 h, (5) *Staphylococcus* spp. on Baird Parker agar (Condalab) supplemented with egg yolk tellurite (Condalab) at 37 °C for 48 h, (6) *Enterococcus* spp. on Kanamycin Aesculin Azide confirmatory agar (Condalab) at 37 °C for 48 h, (7) *Clostridium* spp. on Tryptose Sulfite Cycloserine agar (Condalab) supplemented with egg yolk tellurite (Condalab) at 37 °C for 48 h anaerobically (Anaerobic Jar 2.5 L, Anaerocult A system), (8) LAB (Gram positive) on MRS agar (Condalab) at 37 °C for 72 h anaerobically (Anaerobic Jar 2.5 L, Anaerocult A system), and (9) *Bifidobacterium* spp. on TOS Propionate agar (Condalab) supplemented with Lithium-Mupirocin (MUP) (Condalab) at 37 °C for 48 h anaerobically (Anaerobic Jar 2.5 L, Anaerocult A system). Extended incubations up to 120 h did not result in any additional colonies. The results are expressed as logcfu/g for petri dishes containing 30–300 colonies.

#### 2.5.3. DNA Extraction, PCR Amplification and 16S rRNA Gene Sequencing

Duplicate fecal samples of each group (H_IPC, D_IPC, H_FP, and D_FP) at the beginning and end of the experimental period were subjected to DNA isolation using the NucleoSpin Stool Mini Kit (Macherey-Nagel GmbH and Co. KG, Düren, Germany) and according to the manufacturer’s guidelines. Then, the samples were processed to Next Generation Sequencing (NGS) and specifically to MiSeq Illumina sequencing, which was conducted by “Mrdnalab” (www.mrdnalab.com, Shallowater, TX, USA, accessed on 5 September 2022). Primers that amplify the V1-V3 locus of the bacterial 16S rRNA gene were used (27F/519R, AGRGTTTGATCMTGGCTCAG/GTNTTACNGCGGCKGCTG). PCR conditions and subsequent DNA library preparation have been presented in our previous work [13,16]. Raw data processing was carried out by the pipelines of “Mrdnalab” and data were assigned to Operational taxonomic units (OTUs) after clustering at 3% divergence (97% similarity). Employing BLASTn, OTUs were taxonomically classified to a curated database sourced from NCBI (https://www.ncbi.nlm.nih.gov, accessed on 5 September 2022) and RDPII (http:/www./rdp.cme.msu.edu, accessed on 5 September 2022) and corresponded to taxonomic levels (from kingdom to genus) in “counts” files. After organizing the OTUs “counts” file in the appropriate format [18], further analysis of OTU-level data along with the calculation of diversity indices was conducted via the R platform and Rhea scripts developed by Lagkouvardos et al. [18].

### 2.6. Stool SCFAs and Lactic Acid Profile

Stool short-chain fatty acids (SCFAs) and lactic acid extraction, purification, and quantification were carried out according to Prapa et al. [19]. Levels of SCFAs and lactic acid were determined using an High-Performance Liquid Chromatography (HPLC) system (Shimadzu Corp., Duisburg, Germany) working on LabSolutions™ software (Shimadzu Corp.), consisting of a Nucleogel ION 300 OA column (Macherey-Nagel), a DGU-20A5R degassing unit, an LC-20AD pump, a CTO-20AC oven at 85 °C, and a RID-10A refractive index detector.

Stool SCFAs and lactic acid concentrations were expressed as mean μmol/g of feces, using the equation [20]:

$$\mathrm{SCFAs}\mathrm{and}\mathrm{lactic}\mathrm{acid}\;(\mu mol/g)=\frac{\mathrm{organic}\mathrm{acid}\mathrm{in}\mathrm{fecal}\mathrm{contents}\left(\frac{\mathrm{mmol}}{\mathrm{mL}}\right)\times d\left(mL\right)\times 1000}{\mathrm{weight}\mathrm{of}\mathrm{fecal}\mathrm{sample}\;(g)}$$

where Vd represents the total volume of dilution of the stool samples.

### 2.7. Statistical Analysis

The data are expressed as mean value ± standard error of the mean (SEM) or mean value ± standard deviation (STDEV). Statistical analysis was performed with Statistica v.12 software (StatSoft, Inc., Tulsa, OK, USA). A one-way ANOVA coupled with the Bonferroni post-hoc test was applied to assess differences in inflammatory markers, SCFAs, and cecal (fluid and tissue) microbiota. Factorial ANOVA followed by the Bonferroni post-hoc test was applied to assess differences in immobilized and free cell levels, blood markers, body weight, stool microbiota populations, and microbiome abundances. Statistical significance was set at *p* < 0.05.

## 3. Results and Discussion

Novel wild-type strains that hold potential probiotic properties after in vitro evaluation need to be tested in vivo, as a following step, to confirm promotion of gut homeostasis. *P. acidilactici* ORE5, previously isolated from kefir grains, has exerted presumptive probiotic properties in vitro, whereas immobilized *P. acidilactici* ORE5 cells on pistachio nuts were successfully incorporated in soft cheese, indicating quality improvement and prolongation of the product’s shelf-life [14]. On the other hand, Corinthian currant supplementation for 4 weeks in STZ-induced diabetic rats served as a functional dietary component; the dietary intervention had a beneficial effect on enterococci counts and enhanced the presence of cecal LAB [13]. Given these preliminary studies of our group, *P*. *acidilactici* ORE5 was chosen as a potential probiotic culture, and the immobilized cells on Corinthian currants were then assessed as a functional ingredient for the regulation of the gut microbiome in STZ-induced diabetic rats.

### 3.1. Immobilized P. acidilactici ORE5 Cells on Corinthian Currant

**Figure 2:**
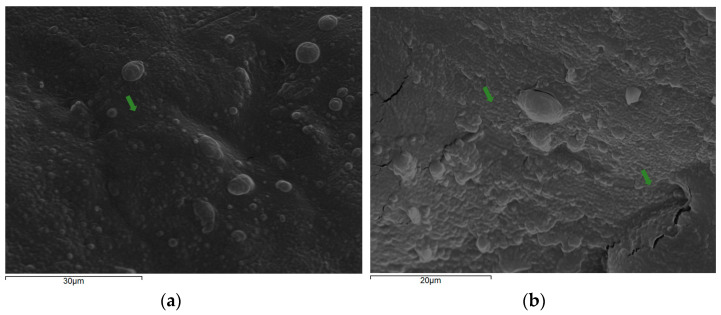
Electron micrographs of immobilized *P. acidilactici* ORE5 cells on Corinthian currants on 30 μm (**a**) and 20 μm (**b**) scale. Cells are shown with arrows.

Initially, the optimization of cell immobilization conditions was studied by evaluating the effect of the duration of the process and the ratio of food carrier/cell culture on cell loads. Immersing Corinthian currants at an 80% ratio in the immobilization mixture for 6 h was chosen as the optimum condition, considering the higher (*p* < 0.05) cell loads determined and the requirements of the food industry for rapid and cost-effective processes. Of note, indicative micrographs of the immobilized *P. acidilactici* ORE5 on Corinthian currants are presented in Figure 2.

Subsequently, the effect of freeze-drying on cell viability of free or immobilized *P. acidilactici* ORE5 cells on Corinthian currant was assessed, and the results are presented in Table 1. Considering the cell loads of wet free and immobilized cells at day 0, it is estimated that approximately 1 logcfu/g is lost, corresponding to 10% cell loss. Freeze-drying led to a significant (*p* < 0.05) decrease of cell loads of both free and immobilized cells compared to wet cells. However, the survival rates of immobilized cells were higher (*p* < 0.05) than free cells (99.65 and 98.43%, respectively), indicating the protective effect of cell immobilization. The positive effect of cell immobilization on the viability of cells during the freeze-drying process has also been documented previously [11,21,22].

The production of immobilized *P. acidilactici* ORE5 cells on Corinthian currants followed by freeze-drying led to cell concentrations over the minimum levels recommended by the International Probiotics Association Europe and The International Scientific Association for Probiotics and Prebiotics (>7 logcfu/g, Table 1) [23]. Specifically, cell concentrations were equal to 3.90 × 0^8^ cfu/g at day 0 and to 3.20 × 10^8^ cfu/g at day 15. Thus, daily supplementation with 2.2 g of immobilized cells on Corinthian currants (representing the 10% *w*/*w* of the diet) resulted in a dosage equal to ~10^9^ cfu. Of note, fresh immobilized *P. acidilactici* ORE5 cells on Corinthian currants were prepared each week to ensure stability of the concentration throughout the experimental period.

The nature of the culture (wet or freeze-dried), as well as the storage temperature (20 °C or 4 °C) significantly (*p* < 0.05) affected the viability of both free and immobilized cells. Storage at 4 °C resulted in higher cell loads and survival rates (%) compared to storage at RT (*p* < 0.05). During storage at RT, a significant (*p* < 0.05) decrease in cell loads of all cultures (wet or freeze-dried, free or immobilized) was observed at 15 and 30 days. In specific, they ranged at levels lower than the minimum recommended cell concentration (7 logcfu/g) after 30 days of storage. Of note, wet cultures concentration was lower (*p* < 0.05) compared to freeze-dried cultures, while the highest cell loads were recorded in freeze-dried immobilized cells (6.54 ± 0.02 logcfu/g). Storage at refrigerated temperature for 30 days resulted in higher (*p* < 0.05) cell loads (>7.40 logcfu/g) compared to storage at 20 °C, in all cases. Immobilized cells on Corinthian currants remained stable after 15 days of storage at 4 °C (*p* > 0.05 compared to day 0), but cell loads were significantly decreased after 30 days of storage (*p* < 0.05). Freeze-fried immobilized *P. acidilactici* ORE5 cells on Corinthian currants exhibited the highest survival rates (96.39%), while the corresponding cell survival of freeze-dried free cells was 86.99% after 30 days of storage. The results are in accordance with a similar study [11], at which immobilized cells of other LAB strains on raisins were decreased compared to immobilized cells on other supports, such as pistachios or cereals. This outcome can be attributed to the polyphenols present in Corinthian currants and the structure and texture of the fruit (soft and chewy), owing to the drying manufacturing process; however, they retained a high moisture content, which leading to a slightly plump and juicy ingredient.

Probiotics are generally consumed as pill-type products; however, their incorporation in food ingredients or food systems could enhance their use [11,24]. Immobilization of probiotic cells on natural food ingredients has been suggested as a method to preserve cell viability during storage [11] and through the passage via the gastrointestinal tract [10]. Therefore, functional cultures can reach their point of action (which is mainly the large intestine) and exert the potential health effects. Similar results were also recently reported by our group [10].

### 3.2. Body Weight, Biochemical Profile and Inflammatory Factors

Following the production of the functional ingredients, the potential alterations in the gut microbiome composition of STZ-induced diabetic rats after a 4-week dietary intervention were examined. Rats received immobilized cells of *P. acidilactici* ORE5 on Corinthian currants or free cells in a concentration of ~10^9^ cfu/day. A similar daily dosage of probiotics is reported elsewhere [25,26] and is in accordance with the International Scientific Association for Probiotics and Prebiotics [20].

Body weight and blood measurements revealed significant differences among the groups that could be attributed to the STZ-induced diabetic state and to the dietary intervention with free or immobilized cells on Corinthian currants.

**Figure 3:**
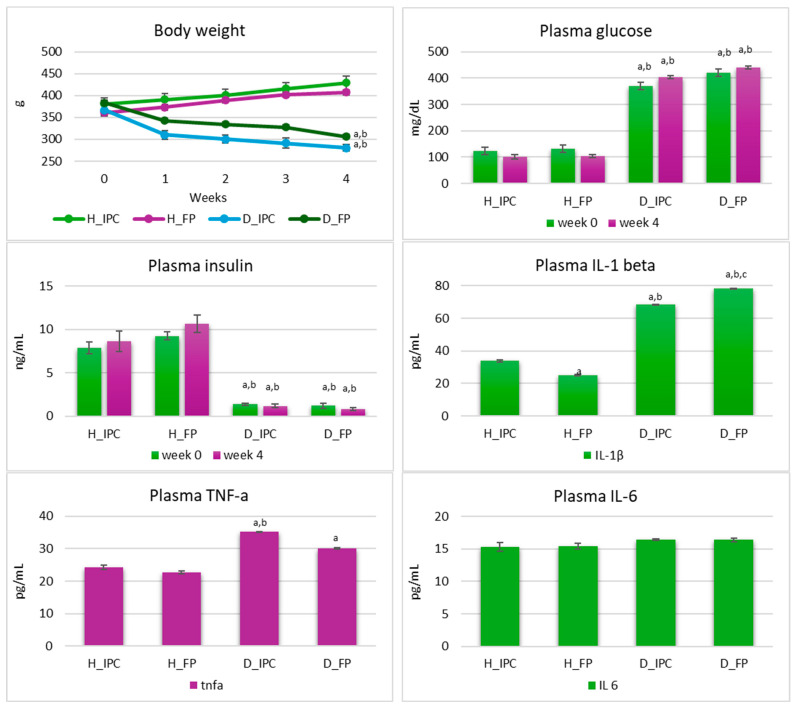

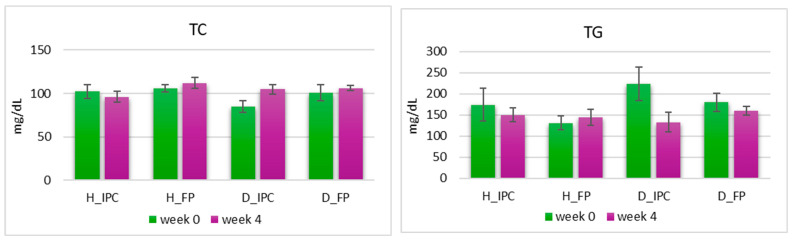
Body weight and plasma glucose, insulin, TNF-a, IL-1 beta, IL-6, total cholesterol (TC), and triglycerides (TG) levels in healthy and STZ-induced diabetic rats during the dietary intervention with free or immobilized *P. acidilactici* ORE5 cells on Corinthian currants. Data are expressed as mean values ± SEM (*n* = 6 per group). Statistically significant differences are highlighted with different letters: ^a^ *p* < 0.05 vs. group H_IPC, ^b^ *p* < 0.05 vs. group H_FP, and ^c^ *p* < 0.05 vs. group D_IPC. H_IPC: healthy animals that received the control diet supplemented with freeze-dried immobilized *P. acidilactici* ORE5 cells on Corinthian currants, D_IPC: diabetic animals that received the control diet supplemented with freeze-dried immobilized *P. acidilactici* ORE5 cells on Corinthian currants, H_FP: healthy animals that received the control diet supplemented with free *P. acidilactici* ORE5 cells, D_FP: diabetic animals that received the control diet supplemented with free *P. acidilactici* ORE5 cells.

In diabetic groups, progressive weight loss was observed (Figure 3). Specifically, after 4 weeks, in groups D_IPC and D_FP, body weight was lower compared to baseline values (*p* < 0.05) and compared to healthy animals (*p* < 0.05 vs. H_IPC and H_FP). In rats with STZ-induced T1DM, progressive loss of body weight is a common observation [27]. STZ selectively destroys the insulin-producing beta cells in the pancreas, mimicking the autoimmune destruction seen in human T1DM. With the destruction of beta cells, insulin production is decreased or absent. In the absence of insulin, cells are unable to effectively utilize glucose for energy production, leading to a catabolic state where tissue breakdown occurs to meet energy demands, including muscle and fat, thus resulting in weight loss. Dietary interventions can play a significant role in managing the progressive weight loss associated with T1DM [28]. While these interventions may not reverse the underlying cause of the disease (insulin deficiency), they can help improve overall health [29] and potentially mitigate some of the complications associated with diabetes. However, in the present study, the dietary intervention with free or immobilized cells on Corinthian currants did not affect the progressive loss of body weight in STZ-induced diabetic rats. Similar results have been reported in the literature [30], indicating that functional foods or ingredients could contribute to the management of the disease in other ways (e.g., moderating inflammation or causing microbiome alterations) [31,32].

Plasma glucose levels were significantly increased in the diabetic groups compared to the healthy groups (*p* < 0.05) at baseline, as well as in the 4th week of the study, and the dietary intervention had no effect (*p* > 0.05, Figure 3). On the other hand, insulin levels were significantly lower in diabetic groups compared to the healthy animals (*p* < 0.05) at both timepoints and were not influenced by the dietary regimen (*p* > 0.05). In T1DM, where insulin production is severely impaired due to the destruction of pancreatic beta-cells, dietary interventions are not sufficient to normalize insulin or glucose levels, but can play a supportive role in managing and mitigating the complications associated with the disease. While dietary modifications can complement insulin therapy and help optimize blood glucose management in T1DM, they are not considered a substitute for insulin therapy. Hence, insulin administration remains the cornerstone of treatment for T1DM in both animals and humans [33].

Inflammation status is of great importance in the emergence, development, and management of T1DM. In this vein, levels of inflammatory factors TNF-a, IL-1 beta, and IL-6 were explored after 4 weeks of the dietary intervention. Plasma IL-6 levels were similar in all groups (*p* > 0.05). In diabetic groups that received the immobilized cells, TNF-a levels were higher compared to both healthy groups (*p* < 0.05 vs. H_IPC and H_FP). In diabetic groups that received the free cells, TNF-a levels were increased compared to healthy groups that received the same diet (free cells) (*p* < 0.05), while ranging in similar levels between the diabetic groups (*p* > 0.05). Furthermore, levels of IL-1 beta were higher in both diabetic groups compared to healthy animals (*p* < 0.05). According to Wang-Fischer and Garyantes [34], increased levels of plasma cytokines, specifically TNF-a, IL-4, and IL-6, constitute strong evidence of the inflammatory state in the STZ-induced diabetic model. Of note, levels of IL-1 beta were significantly lower in diabetic rats that received the immobilized *P. acidilactici* ORE5 cells on Corinthian currants compared to diabetic rats that received the free cells (*p* < 0.05). In our previous study [13], the dietary intervention with Corinthian currants in STZ-induced diabetic rats resulted in lower levels of IL-1 beta in diabetic rats compared to groups that received the control diet, as well. Taken together these results, an anti-inflammatory potential of Corinthian currants is suggested, which was also noticed when, rich in polyphenols, Corinthian currants were used as a functional food constituent and an immobilization carrier for presumptive probiotic cells. In line with these results, a reduction in IL-1 beta was witnessed due to *Lactobacillus rhamnosus* EM1107 administration to STZ-induced diabetic rats (10^9^ cfu/day, for 30 days), suggesting an immune-inflammatory efficacy of the strain [35].

At the beginning and end of the study, blood biochemical parameters, including TC and TG, were also determined. TC and TG were neither affected by the condition of the disease nor by the dietary intervention and ranged at similar levels in all groups and at both timepoints (*p* > 0.05). Similar findings are also reported elsewhere [16].

### 3.3. Fecal and Intestinal Microbiota Analysis

**Figure 4:**
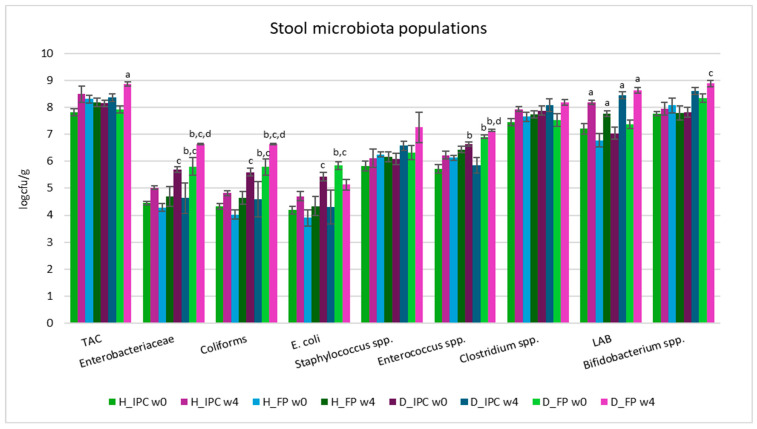
Stool microbiota populations in healthy and STZ-induced diabetic rats during the dietary intervention with free or immobilized *P. acidilactici* ORE5 cells on Corinthian currants. Data are expressed as mean values ± SEM (*n* = 6 per group). Statistically significant differences are highlighted with different letters: ^a^ *p* < 0.05 vs. baseline values of the same group, ^b^ *p* < 0.05 vs. group H_IPC, ^c^ *p* < 0.05 vs. group H_FP, and ^d^ *p* < 0.05 vs. group D_IPC. H_IPC: healthy animals that received the control diet supplemented with freeze-dried immobilized *P. acidilactici* ORE5 cells on Corinthian currants; D_IPC: diabetic animals that received the control diet supplemented with freeze-dried immobilized *P. acidilactici* ORE5 cells on Corinthian currants; H_FP: healthy animals that received the control diet supplemented with free *P. acidilactici* ORE5 cells; D_FP: diabetic animals that received the control diet supplemented with free *P. acidilactici* ORE5 cells; w0: week 0, w4: week 4, TAC: Total Aerobic Counts, LAB: lactic acid bacteria.

According to the results of the microbiological analyses, at the baseline of the dietary intervention, significant differences in fecal microbial populations were observed between healthy and animal T1DM models (Figure 4). Increased levels of *Enterobacteriaceae*, coliforms, *E. coli*, and *Enterococcus* spp. were noted in the feces of diabetic rats compared to the healthy animals. More specifically, at baseline, *Enterobacteriaceae,* coliform, and *E. coli* loads in the H_IPC group were lower compared to D_FP animals (*p* < 0.05), and in the H_FP group they were lower than in the D_FP and D_IPC groups (*p* < 0.05). In group H_IPC, *Enterococcus* spp. was decreased compared to D_IPC and D_FP animals (*p* < 0.05). The changes in the composition of fecal populations following the injection of STZ could be potentially connected to the onset and progression of T1DM, highlighting the interplay between gut dysbiosis (expressed as an imbalanced microbiome and increased presence of pathogens) and intestinal inflammation, as also reported previously [36,37,38].

After 4 weeks of the dietary intervention with free *P. acidilactici* ORE5 cells, TAC levels were increased in the D_FP group (*p* < 0.05 compared to baseline values) and significantly increased levels (*p* < 0.05) of *Enterobacteriaceae* and coliforms were recorded compared to healthy groups and to diabetic animals receiving the immobilized cells, where the corresponding values were similar to the levels of healthy animals (*p* > 0.05). Additionally, in the D_FP group, levels of *Enterococcus* spp. were further elevated after 4 weeks (*p* < 0.05 compared to H_IPC and D_IPC), while in the D_IPC group the levels were restored to the values of the healthy groups (*p* > 0.05). Same results were observed in our previous study [13], where the dietary intervention with Corinthian currants in STZ-induced diabetic animals resulted in normalized *Enterococcus* spp. counts (to the levels of healthy groups), while in the diabetic groups that received the control diet increased cell counts were documented. This result might be attributed to the components of Corinthian currants, and their action is maintained when they are used as immobilization agents of the presumptive probiotic cells.

Counts of *Bifidobacterium* spp. were significantly elevated in the D_FP group compared to the H_FP group (*p* < 0.05) after 4 weeks. LAB counts were elevated in all groups compared to baseline values (*p* < 0.05), indicating the survival of the administrated strain (*P. acidilactici* ORE5). Of note, in our previous study, where Corinthian currants were administered to STZ-induced diabetic rats, no such differences were noticed [13]. Given the differences in microbiota composition mapped in groups receiving the free or immobilized cells, a possible synergistic effect of the functional food ingredient comprised of the presumptive probiotic strain and the Corinthian currants could be implied, but further experiments should be designed to validate such a hypothesis.

In line with our results, the supplementation with the synbiotic formula “FloraGuard” (containing inulin and probiotics) in healthy rats for 8 weeks led to increased *Lactobacillus* and *Bifidobacteria* counts, while coliforms levels were significantly reduced [39]. In a similar study, significantly increased LAB counts after 30 days of supplementation with *L. rhamnosus* BSL and *L. rhamnosus* R23 were reported [40], while the administration of *Lactobacillus kefiranofaciens* M and *Lactobacillus kefiri* K (10^8^ cfu/day for 8 weeks) in STZ-induced diabetic mice led to a balanced intestinal microbiota by enhancing the LAB and bifidobacteria presence and downregulating the opportunistic pathogen *Clostridium perfringens* counts [41].

Similar results were observed in the levels of the microbial populations in cecum tissue and cecal content samples (Table 2), where different microbial concentrations were recorded in all groups depending on the health status (*p* < 0.05) and the dietary regimen (*p* < 0.05).

Regarding the cecum tissue adherent populations, diabetic animals that received free *P. acidilactici* ORE5 cells exerted higher levels of *Enterobacteriaceae*, coliforms, *E. coli*, *Staphylococcus* spp., and *Enterococcus* spp. compared to both healthy groups (*p* < 0.05) and to the diabetic animals that received the immobilized cells (*p* < 0.05 for all populations except *Staphylococcus* spp.). On the other hand, these populations were rendered to normal values in the D_IPC group, where fewer differences were observed compared to the healthy animals. Particularly in the D_IPC group, staphylococci were higher compared to the H_IPC and H_FP groups (*p* < 0.05), yet *Enterobacteriaceae* and *E. coli* were higher compared to the H_FP group (*p* < 0.05), but similar to the H_IPC animals (*p* > 0.05). No differences were noticed in coliforms in H_IPC and H_FP groups (*p* > 0.05). *Clostridium* spp. was increased in the D_FP group compared to the H_FP animals (*p* < 0.05), but the levels were similar to those in the H_IPC group (*p* > 0.05). The highest levels of *Bifidobacterium* spp. were recorded in the D_IPC group (*p* < 0.05 compared to all groups), and LAB counts were higher in the H_IPC group compared to H_FP animals (*p* < 0.05), but similar between the D_IPC and D_FP groups.

In the cecal content (portion of the intestinal lumen with the highest microbial populations associated with fecal microbiota), significant differences were observed between the T1DM animals and the healthy groups and between groups that received the free or the immobilized cells (Table 2). Lower TAC levels were observed in both diabetic groups compared to the H_IPC group; TAC counts were elevated compared to the H_FP group (*p* < 0.05); and TAC of H_IPC animals was increased compared to the H_FP group (*p* < 0.05). In diabetic rats that received the immobilized cells on Corinthian currants (D_IPC), higher levels of *Enterobacteriaceae* and *E. coli* were noted compared to the H_FP group (*p* < 0.05), but they ranged in similar levels with the H_IPC and D_FP groups (*p* > 0.05). Also, in D_IPC animals, higher levels of coliforms were encountered compared to both healthy groups (*p* < 0.05), whilst *Staphylococcus* spp. was lower compared to the H_IPC group and higher than H_FP animals (*p* < 0.05). *Enterococcus* spp. and LAB (of the D_IPC group) were significantly reduced compared to H_IPC animals (*p* < 0.05).

In STZ-induced diabetic rats that received the free cells, levels of *Enterobacteriaceae* and *E. coli* were higher compared to the H_FP group (*p* < 0.05), but normalized to counts of the H_IPC group (*p* > 0.05). Coliforms of D_FP animals were significantly elevated compared to the H_FP group and ranged in lower levels compared to D_IPC animals (*p* < 0.05). In the same group (D_FP), staphylococci and enterococci counts were lower than H_IPC animals (*p* < 0.05), and staphylococci levels were also higher than the H_FP group (*p* < 0.05). *Bifidobacterium* spp. concentration was the lowest in the D_FP group compared to all groups (*p* < 0.05 vs. H_IPC, H_FP, and D_IPC), and LAB counts were higher in the D_FP than H_FP and D_IPC groups (*p* < 0.05).

In a similar study, the supplementation of *Bacillus coagulans* spores (10^9^ spores/day for 30 days) either alone and/or with inulin in healthy rats resulted in an increase of LAB counts in feces, cecum tissue, and distal segments of the gastrointestinal tract. Higher levels of *Lactobacillus* spp. were observed throughout the small intestine of the synbiotic and prebiotic fed rats compared to the probiotic fed and control diet groups, and significantly decreased *Enterobacteriaceae* counts in the cecum and colon tissue samples of the synbiotic, probiotic, and prebiotic supplemented groups were recorded [42], underlying the different response of the microbial populations in the intestine and the effect of the diet administered.

### 3.4. Microbiome Alterations Using NGS of 16S rRNA Gene

Fecal microbiome abundances were determined by NGS based on the 16S rRNA gene sequence and analyzed using appropriate bioinformatic tools. The results of the NGS analysis revealed that different changes occurred in the microbiome composition of STZ-induced diabetic rats compared to the healthy rats, depending on the form of the cells that the animals received.

**Figure 5:**
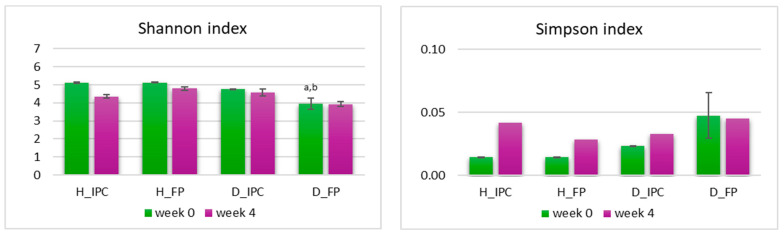
A-diversity indices Simpson’s and Shannon’s level after the dietary intervention with free or immobilized *P. acidilactici* ORE5 cells on Corinthian currants at fecal samples in healthy and STZ-induced diabetic rats, as determined by 16S rRNA gene NGS. Data expressed as mean ± SEM (*n* = 2 per group). Statistically significant differences are highlighted with different letters: ^a^ *p* < 0.05 vs. group H_IPC and ^b^ *p* < 0.05 vs. group H_FP. H_IPC: healthy animals that received the control diet supplemented with freeze-dried immobilized *P. acidilactici* ORE5 cells on Corinthian currants; D_IPC: diabetic animals that received the control diet supplemented with freeze-dried immobilized *P. acidilactici* ORE5 cells on Corinthian currants; H_FP: healthy animals that received the control diet supplemented with free *P. acidilactici* ORE5 cells; D_FP: diabetic animals that received the control diet supplemented with free *P. acidilactici* ORE5 cells.

Firstly, Shannon’s and Simpson’s α-diversity indices were determined (Figure 5). Simpson’s index was neither affected by the STZ-induced diabetes nor by the dietary intervention (*p* > 0.05 between groups). At baseline, in STZ-induced diabetic rats (D_FP group) a significantly lower Shannon’s index value compared to both healthy groups (*p* < 0.05 vs. H_IPC and H_FP) was documented, but such a difference was not observed in the D_IPC group (*p* > 0.05 compared to D_FP and to H_IPC and H_FP). After 4 weeks, no significant differences were mapped (*p* > 0.05). In accordance with our results, decreased α-diversity has been observed in STZ-induced diabetic rats (STZ dosage 60 mg/kg) [43] in alloxan-induced T1DM rats [44] and in T1DM children [45] and has been associated with gut dysbiosis that occurs in the disease [46]. However, in autoimmune T1DM in humans, the causal relationship between the intestinal dysbiosis and the onset of the disease remains under investigation.

At phylum level and after 4 weeks of the dietary intervention, in the STZ-induced groups (D_IPC and D_FP), a significant increase of Actinobacteria abundance was observed compared to baseline and to healthy animals (*p* < 0.05, Table 3). A significantly elevated percentage of Verrucomicrobia was also observed in diabetic animals that were administered with the immobilized cells on Corinthian currants (D_IPC) compared to healthy groups (*p* < 0.05).

**Figure 6:**
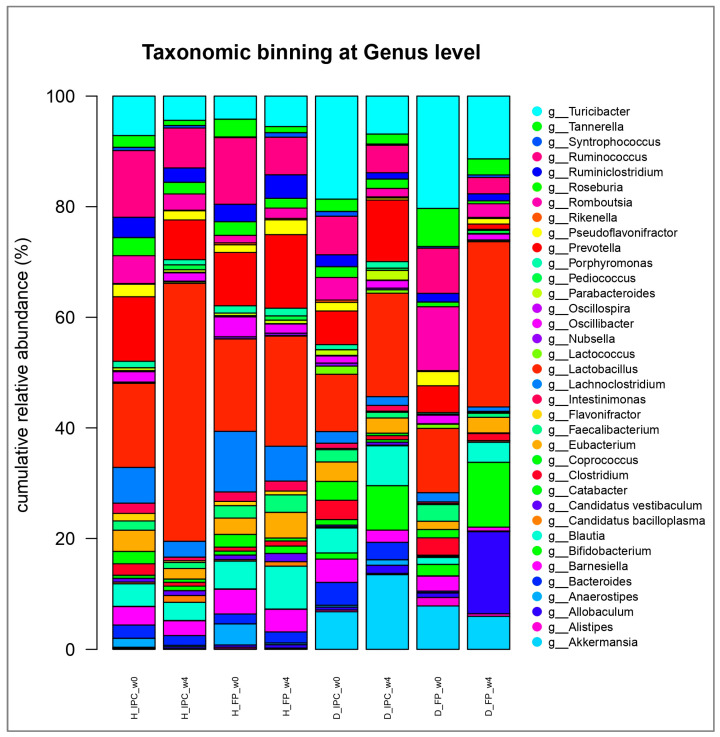
Normalized relative abundances (%) at genus taxonomic level after the dietary intervention with free or immobilized *P. acidilactici* ORE5 cells on Corinthian currants at fecal samples in healthy and STZ-induced diabetic rats, as determined by 16S rRNA gene NGS. Data are expressed as mean values (*n* = 2 per group). H_IPC: healthy animals that received the control diet supplemented with freeze-dried immobilized *P. acidilactici* ORE5 cells on Corinthian currants; D_IPC: diabetic animals that received the control diet supplemented with freeze-dried immobilized *P. acidilactici* ORE5 cells on Corinthian currants; H_FP: healthy animals that received the control diet supplemented with free *P. acidilactici* ORE5 cells; D_FP: diabetic animals that received the control diet supplemented with free *P. acidilactici* ORE5 cells; w0: week 0; w4: week 4.

At the genus level, several significant shifts were observed between the groups and over time. At baseline, decreased abundance of the genus *Flavonifractor* was detected in the diabetic groups (D_IPC and D_FP) compared to the healthy animals (H_IPC) (*p* < 0.05, Figure 6). After 4 weeks, increased levels of *Akkermansia* and *Bifidobacterium* were noted in animals that received the immobilized *P. acidilactici* ORE5 cells on Corinthian currants compared to the healthy groups and to baseline values (*p* < 0.05 vs. H_IPC, H_FP, and vs. baseline). Likewise, increased abundance of *Bifidobacterium* (*p* < 0.05 vs. H_IPC, H_FP, and vs. baseline) and *Allobaculum* (*p* < 0.05 vs. H_IPC, H_FP, D_IPC, and vs. baseline) were documented in diabetic rats that followed the diet enriched with the free cells of the presumptive probiotic *P. acidilactici* ORE5 strain. Similarly, an increased abundance of *Lactobacillus* genus was noticed in healthy rats receiving the immobilized culture compared to baseline values (*p* < 0.05). Finally, in all groups (diabetic or healthy that received free or immobilized cells), *Pediococcus* genus was increased (*p* < 0.05) compared to the initial values.

In our previous study [13], the dietary intervention with Corinthian currants led to an increased abundance of *Candidatus bacilloplasma* in healthy groups, while no significant changes were reported in diabetic groups that received the Corinthian currants. Yet, this implies that changes mapped in this study are probably due to the dietary intervention with the functional ingredient or the free presumptive probiotic cells.

Decreased *Flavonifractor* abundance has been observed in T1DM compared to healthy children, according to Liu et al. [47]. It was suggested that *Flavonifractor plautii* participates in regulating blood glucose metabolism and it has been associated with the attenuation of inflammation in adipose tissue [48], while its abundance in fecal samples could be proposed as a biomarker of health status [46,49]. This genus is mainly (or solely) comprised of *F. plautii* species members of the *Ruminococcaceae* family, which are described as commensal members of human and animal gut microbiota [50].

Increased *Pediococcus* abundance in all groups (STZ-induced diabetic and healthy animals) can be attributed to the survival of the strain (in free or immobilized form), in agreement with the results of a dietary intervention to humans with a probiotic yogurt containing *P. acidilactici* GR-1 cells, where the relative abundance of the species was significantly increased after 12 weeks [51]. Thus, it is not unreasonable to suggest that the increased abundance of the *P. acidilactici* species highlights the successful survival and possible colonization of our strain [52], as well as the effectiveness of the supplementation duration.

The *Allobaculum* genus is currently comprised of three species (*Allobaculum fili*, *Allobaculum mucilyticum*, and *Allobaculum stercoricanis*) that are butyrate producers of the commensal microbiome [53]. The role of these bacteria in inflammatory processes [54] and aging [55] has been explored, while *Allobaculum* abundance can be altered after dietary interventions [56,57]. Increased *Allobaculum* abundance was observed in another study in which *L. casei* CCFM419 was administrated to STZ-induced high-fat diet-fed diabetic C57BL/6J mice [58]. The specific activity of these bacteria towards gut homeostasis and their potential as beneficial target microorganisms are yet to be clarified.

*Akkermansia* genus belongs to the Verrucomicrobia phylum. In particular, the species *Akkermansia muciniphila* is characterized as a next-generation probiotic, while pasteurized cells were approved for use by the European Food Safety Authority, in 2021 [59]. The activity of this species has been examined vastly in obesity, Type-2 Diabetes Mellitus (T2DM), and related diseases and it is suggested that it improves metabolism (glucose homeostasis), alleviates inflammation, enhances intestinal barrier function, and maintains microbiome homeostasis [60]. Positive effects in autoimmunity and delayed diabetes development in non-obese diabetic mice (T1DM) were reported after administration of *A. muciniphila* [61]. Specifically, *A. muciniphila* orally administered in STZ-induced T2DM rats for 4 weeks resulted in significantly increased α-diversity and it was concluded that it improved liver function, reduced gluco-/lipotoxicity, alleviated oxidative stress, suppressed inflammation, and normalized the gut microbiome, thereby ameliorating T2DM [62]. Increased presence (compared to healthy groups) of this commensal and beneficial genus after 4-weeks of the dietary intervention with immobilized *P. acidilactici* ORE5 cells on Corinthian currants could be perceived as a positive result of our study.

Genera, such as *Lactobacillus* and *Bifidobacterium*, are among the most significant probiotic bacteria. They function by regulating the pH of the gut environment, improving barrier function through increased mucus production, releasing antimicrobial peptides, and altering the composition of the gut microbiota [63]. Probiotic lactobacilli can exert their effects on the host in several ways, including the antagonistic activity against pathogens, the amelioration of intestinal barrier function, immunoregulation and anti-inflammatory actions, anticancer/antiproliferative activity, and metabolic regulation [64], while it must be noted that these activities are strain specific. The elevated *Lactobacillus* and *Bifidobacterium* abundances can be interpreted as a beneficial outcome of the study. Similar results with increased abundances of beneficial genera are reported in other works as well, employing probiotics administered in high-fat diet-fed mice [65,66] and in non-obese diabetic mice (T1DM) [67].

### 3.5. Stool SCFAs and Lactate

The concentrations of fecal SCFAs (acetic, propionic, butyric, isobutyric, valeric, and isovaleric acids) and lactic acid were determined in the stool samples in the 4th week of the study (Table 4). According to the results, the concentration of lactic acid was increased (*p* < 0.05) in diabetic animal models that received the immobilized *P. acidilactici* ORE5 cells, compared to the H_IPC group. Moreover, acetic acid was significantly increased in both diabetic groups compared to the healthy animals (*p* < 0.05 vs. H_IPC and H_FP). No significant differences between the different groups (*p* > 0.05) were noted for the rest fatty acids.

Increased concentration of lactic acid could be due to the metabolism of the immobilized *P. acidilactici* ORE5 cells in the intestine. In a similar study, the consumption of probiotics (by STZ-induced mice for 8 weeks) led to increased fecal butyric acid, but other SCFAs were not affected [41]. Results from our previous work in STZ-induced T1DM rats that followed a dietary intervention with Corinthian currants revealed increased concentrations of acetic acid in diabetic groups and elevated amounts in diabetic groups that received the Corinthian currants (compared to diabetic groups that received the control diet) [13]. Elevated levels of acetic acid can be associated with the increased presence of *Bifidobacterium* and *Akkermansia* [68]. An increase in *Bifidobacterium* and *Lactobacillus* genera along with increased cecal and plasma acetate levels and improved glucose tolerance was associated with supplementation of *Bifidobacterium animalis* subsp. *lactis* GCL2505 in mice [65]. In a human trial, the consumption of probiotics contributed to glycemic control and led to increased fecal acetic acid [69].

### 3.6. Limitations, Future Perspectives and Summary of Findings

Several differences arise in our findings when compared with other similar studies; this could be due to the variation of the disease induction (alloxan/STZ models, dosage of the chemical reagent), age of the animals, different dosage of the functional culture (cfu/day), duration of the study, and microbiome/microbiota analysis protocols used (NGS, PCR, plate count, etc.) [44]. According to a recent review [70] concerning a meta-analysis of clinical trials examining the probiotics use in T1DM patients, the fasting blood glucose was decreased after a probiotic intake, while no effects on other markers (serum hemoglobin A1c (HbA1c), C-peptide, and insulin) were observed. However, evidence is limited and more trials have to be conducted to address how probiotic supplementation could be used as a complementary therapeutic strategy in T1DM.

According to the authors’ knowledge, this is the first study that evaluates the effects of a dietary intervention with an immobilized wild-type presumed probiotic strain in T1DM animal models. A limitation of the study was that T1DM occurs naturally in humans as an autoimmune response and therefore the pathogenicity of the disease is different compared to STZ-induced diabetes in rat animal models. Hence, clinical trials in humans should be considered as the next research step to verify the effectiveness of functional ingredients. This approach will elucidate the underlying mechanisms of action necessary for developing novel dietary patterns and therapeutic strategies for the prevention and management of diabetes-related complications and for improving metabolic health in T1DM. Another limitation was that for the microbiome analysis based on the 16S rRNA gene duplicate samples were used; however, significant differences were mapped following previous studies from our group [13,16]; yet more samples could help to shape more safe conclusions and reveal more alterations owing to the probiotic consumption.

To sum up, the first step of our study was the development of freeze-dried immobilized *P*. *acidilactici* ORE5 cells on Corinthian currants and immobilization was confirmed using electron microscopy. The strain exhibited a high survival rate (96.39%) after storage at 4 °C for 30 days. STZ-induced diabetic rats exhibited high counts of fecal enterococci, *Enterobacteriaceae*, coliforms, and *E. coli*, decreased *Flavonifractor* abundance, and lower a-diversity compared to healthy groups. After a 4-week dietary intervention with free or immobilized cells, increased OTUs of Actinobacteria phylum and *Bifidobacterium* genus were detected in both diabetic groups, while fecal acetic acid was higher compared to healthy animals. The consumption of the functional ingredient (immobilized cells on Corinthian currants) by STZ-induced diabetic animals led to significantly lower levels of plasma IL-1 beta compared to diabetic groups that received a diet rich in free *P. acidilactici* ORE5 cells, a finding that suggested a possible anti-inflammatory action. Furthermore, lower or restored fecal and cecal enterococci, *Enterobacteriaceae*, coliforms, and *E. coli* were detected, and increased LAB counts along with elevated Verrucomicrobia and *Akkermansia* OTUs were recorded, while fecal lactic acid was increased in diabetic rats that received the immobilized cells. An increase in *Lactobacillus* genus was also observed in healthy animals that received the immobilized cells of *P. acidilactici* ORE5 on Corinthian currants, and *Pediococcus* was increased in all groups, underlying the survival of the administrated strain.

## 4. Conclusions

The aim of our study was to explore possible microbiome alterations that might occur in a STZ-induced T1DM animal model after a 4-week dietary intervention with free or immobilized cells of the presumed probiotic *P. acidilactici* ORE5 strain on Corinthian currants. The effects on other parameters (biochemical and inflammatory factors) related to the disease were also examined. STZ-induced diabetes was characterized by gut dysbiosis and lower α-diversity in feces and intestinal microbiome. Administration of free or immobilized *P. acidilactici* ORE5 on Corinthian currants was associated with positive effects on the fecal, cecal tissue-adherent, and cecal microbiota, as well as in the microbiome abundances of selected genera and in fecal lactic acid concentration. Furthermore, lower levels of IL-1 beta in diabetic rats that received the functional food ingredient compared to groups that received the free cells were recorded, indicating a possible anti-inflammatory action. Overall, the results of the present research demonstrated that functional ingredients could ameliorate gut dysbiosis present in T1DM and could be used to design dietary patterns, aiming at T1DM management.

Given the caloric value of Corinthian currants (294 kcal/100 g) and the caloric content of the standard diet of rats (388 kcal/100 g), the daily caloric intake of Corinthian currants was 7% in rats that received the immobilized cells. In humans that receive a daily caloric intake of about 2000 kcal, the 7% corresponds to 140 kcal, equal to 48 g of Corinthian currants, an amount that can be easily consumed daily. However, the limitations involved in extrapolating results from animal models to humans should also be considered. Importantly, a cell load of at least 10^9^ probiotic cells can also be achieved through the administration of the specific number of currants containing the immobilized *P. acidilactici* ORE5 cells.

Finally, it should be noted that well-designed clinical trials are necessary, in order to confirm the beneficial effects in humans. In this vein, possible synergistic effects of personalized dietary interventions with an anti-diabetic treatment should also be explored.

## Figures and Tables

**Table 1:** Effect of immobilization and storage at RT and 4 °C on cell levels and survival rates of free and immobilized *P. acidilactici* ORE5 cells on Corinthian currants.

Storage Temperature	Form	Nature	Logcfu			% Survival	
			**d0**	**d15**	**d30**	**d15**	**d30**
RT	Immobilized	W	8.62 ± 0.05	6.74 ± 0.08	2.30 ± 0.01	78.19 ± 1.80	26.68 ± 0.35
		FD	8.59 ± 0.05	7.75 ± 0.02	6.38 ± 0.01	90.22 ± 0.85	74.27 ± 0.62
	Free	W	9.61 ± 0.05	7.43 ± 0.02	5.09 ± 0.01	77.31 ± 2.02	52.96 ± 0.39
		FD	9.46 ± 0.03	7.62 ± 0.01	6.54 ± 0.02	80.54 ± 0.61	69.13 ± 0.84
4 °C	Immobilized	W	8.62 ± 0.05	8.41 ± 0.02	7.55 ± 0.02	97.56 ± 0.99	87.58 ± 0.88
		FD	8.59 ± 0.05	8.51 ± 0.01	8.28 ± 0.01	99.06 ± 1.01	96.39 ± 0.65
	Free	W	9.61 ± 0.05	8.97 ± 0.01	7.41 ± 0.01	93.34 ± 0.81	77.10 ± 0.55
		FD	9.46 ± 0.03	9.08 ± 0.03	8.23 ± 0.01	95.98 ± 0.64	86.99 ± 0.71

Data are expressed as mean values ± standard deviation (STDEV) (*n* = 3 per sample). Wet and freeze-dried free cells were used as control samples. W: Wet cultures, FD: freeze-dried cultures after rehydration, d0: day 0, d15: day 15, d30: day 30.

**Table 2:** Cecal tissue adherent and cecal content microbiota populations in healthy and STZ-induced diabetic rats after the dietary intervention with free or immobilized *P. acidilactici* ORE5 cells on Corinthian currants.

	Cecum (Tissue Adherent)				Cecal Content			
Group	H_IPC	H_FP	D_IPC	D_FP	H_IPC	H_FP	D_IPC	D_FP
TAC	5.70 ± 0.06	5.42 ± 0.14	6.30 ± 0.26 ^b^	5.88 ± 0.16	8.52 ± 0.11	6.75 ± 0.03 ^a^	7.70 ± 0.25 ^a,b^	7.50 ± 0.03 ^a,b^
*Enterobacteriaceae*	3.15 ± 0.16	2.43 ± 0.17	3.86 ± 0.48 ^b^	5.31 ± 0.10 ^a,b,c^	5.58 ± 0.28	3.43 ± 0.31	6.02 ± 0.01 ^b^	5.29 ± 0.08 ^b^
Coliforms	3.13 ± 0.13	2.40 ± 0.17	3.53 ± 0.56	5.20 ± 0.06 ^a,b,c^	5.57 ± 0.28	3.35 ± 0.15 ^a^	6.28 ± 0.09 ^a,b^	5.29 ± 0.08 ^b,c^
*E. coli*	3.01 ± 0.16	2.33 ± 0.03	3.69 ± 0.51 ^b^	5.25 ± 0.10 ^a,b,c^	5.41 ± 0.32	3.47 ± 0.15	5.74 ± 0.05 ^b^	5.12 ± 0.02 ^b^
*Staphylococcus* spp.	3.02 ± 0.10	2.90 ± 0.07	4.86 ± 0.12 ^a,b^	4.68 ± 0.21 ^a,b^	5.55 ± 0.13	4.52 ± 0.08 ^a^	4.94 ± 0.09 ^a,b^	5.16 ± 0.01 ^a,b^
*Εnterococcus* spp.	3.84 ± 0.05	3.93 ± 0.29	3.87 ± 0.15	4.86 ± 0.19 ^a,b,c^	6.12 ± 0.20	4.85 ± 0.11 ^a^	5.31 ± 0.04 ^a^	4.64 ± 0.04 ^a,c^
*Clostridium* spp.	5.26 ± 0.09	5.00 ± 0.12	5.39 ± 0.12	5.61 ± 0.11 ^b^	6.93 ± 0.30	6.53 ± 0.06	6.55 ± 0.01	6.54 ± 0.01
LAB	5.78 ± 0.07	5.12 ± 0.03 ^a^	6.21 ± 0.28 ^b^	5.34 ± 0.09 ^a^	8.23 ± 0.07	6.28 ± 0.10 ^a^	6.90 ± 0.28 ^a^	7.87 ± 0.15 ^b,c^
*Bifidobacterium* spp.	5.30 ± 0.05	5.53 ± 0.14	6.22 ± 0.05 ^a,b^	5.67 ± 0.19 ^c^	7.61 ± 0.18	7.48 ± 0.02	7.88 ± 0.05	6.71 ± 0.13 ^a,b,c^

Data are expressed as mean values ± SEM (*n* = 6 per group). Statistically significant differences are highlighted with different letters: ^a^ *p* < 0.05 vs. group H_IPC, ^b^ *p* < 0.05 vs. group H_FP, and ^c^ *p* < 0.05 vs. group D_IPC. H_IPC: healthy animals that received the control diet supplemented with freeze-dried immobilized *P. acidilactici* ORE5 cells on Corinthian currants; D_IPC: diabetic animals that received the control diet supplemented with freeze-dried immobilized *P. acidilactici* ORE5 cells on Corinthian currants; H_FP: healthy animals that received the control diet supplemented with free *P. acidilactici* ORE5 cells; D_FP: diabetic animals that received the control diet supplemented with free *P. acidilactici* ORE5 cells; TAC: Total Aerobic Counts; LAB: lactic acid bacteria.

**Table 3:** Normalized relative abundances (%) at phylum taxonomic level after the dietary intervention with free or immobilized *P. acidilactici* ORE5 cells on Corinthian currants at fecal samples in healthy and STZ-induced diabetic rats, as determined by 16S rRNA gene NGS.

	H_IPC		H_FP		D_IPC		D_FP	
Weeks	0	4	0	4	0	4	0	4
Actinobacteria	0.06 ± 0.00	0.0 ± 0.02	0.06 ± 0.02	0.05 ± 0.00	1.12 ± 0.95	8.04 ± 2.02 ^a,b,c^	2.08 ± 0.79	11.73 ± 0.11 ^a,b,c^
Bacteroidetes	22.24 ± 2.64	15.14 ± 4.72	22.71 ± 0.59	24.86 ± 8.61	20.12 ± 3.99	22.49 ± 1.95	16.97 ± 14.47	5.61 ± 0.59
Firmicutes	77.29 ± 2.38	83.51 ± 5.87	76.86 ± 0.59	74.23 ± 7.96	71.75 ± 4.23	55.90 ± 2.38	72.94 ± 9.69	76.42 ± 2.62
Tenericutes	0.30 ± 0.24	1.22 ± 1.18	0.27 ± 0.04	0.80 ± 0.62	0.21 ± 0.01	0.10 ± 0.02	0.16 ± 0.11	0.26 ± 0.09
Verrucomicrobia	0.11 ± 0.02	0.06 ± 0.01	0.10 ± 0.03	0.06 ± 0.02	6.80 ± 0.72	13.46 ± 2.42 ^b,c^	7.85 ± 3.88	5.98 ± 2.01

Data are expressed as mean values ± SEM (*n* = 2 per group). Statistically significant differences are highlighted with different letters: ^a^ *p* < 0.05 vs. baseline ^b^ *p* < 0.05 vs. group H_IPC and ^c^ *p* < 0.05 vs. group H_FP. H_IPC: healthy animals that received the control diet supplemented with freeze-dried immobilized *P. acidilactici* ORE5 cells on Corinthian currants; D_IPC: diabetic animals that received the control diet supplemented with freeze-dried immobilized *P. acidilactici* ORE5 cells on Corinthian currants; H_FP: healthy animals that received the control diet supplemented with free *P. acidilactici* ORE5 cells; D_FP: diabetic animals that received the control diet supplemented with free *P. acidilactici* ORE5 cells.

**Table 4:** Lactic acid and SCFA concentrations (μmol/g) in the stool samples of healthy and STZ-induced diabetic rats after the dietary intervention with free or immobilized *P. acidilactici* ORE5 cells on Corinthian currants.

	Lactic	Acetic	Propionic	Isobutyric	Butyric	Isovaleric	Valeric
H_IPC	2.796 ± 0.170	18.481 ± 0.502	1.382 ± 0.047	0.059 ± 0.006	0.657 ± 0.099	0.058 ± 0.007	0.106 ± 0.012
H_FP	4.014 ± 0.438	26.552 ± 2.610	1.890 ± 0.105	0.559 ± 0.381	1.654 ± 0.522	0.332 ± 0.197	0.160 ± 0.022
D_IPC	6.121 ± 0.783 ^a^	37.280 ± 2.680 ^a,b^	1.899 ± 0.148	0.147 ± 0.101	1.166 ± 0.315	0.046 ± 0.018	0.131 ± 0.016
D_FP	4.307 ± 0.688	38.094 ± 2.712 ^a,b^	1.904 ± 0.188	0.219 ± 0.130	1.005 ± 0.258	0.062 ± 0.024	0.092 ± 0.019

Data are expressed as mean values ± SEM (*n* = 6 per group). Statistically significant differences are highlighted with different letters: ^a^ *p* < 0.05 vs. group H_IPC and ^b^ *p* < 0.05 vs. group H_FP. H_IPC: healthy animals that received the control diet supplemented with freeze-dried immobilized *P. acidilactici* ORE5 cells on Corinthian currants; D_IPC: diabetic animals that received the control diet supplemented with freeze-dried immobilized *P. acidilactici* ORE5 cells on Corinthian currants; H_FP: healthy animals that received the control diet supplemented with free *P. acidilactici* ORE5 cells; D_FP: diabetic animals that received the control diet supplemented with free *P. acidilactici* ORE5 cells.

## Data Availability

The data presented in this study are available in the main article.
